# Selenium Promotes T-Cell Response to TCR-Stimulation and ConA, but Not PHA in Primary Porcine Splenocytes

**DOI:** 10.1371/journal.pone.0035375

**Published:** 2012-04-17

**Authors:** Fei Ren, Xingxiang Chen, John Hesketh, Fang Gan, Kehe Huang

**Affiliations:** 1 Institute of Nutritional and Metabolic Disorders in Domestic Animals and Fowls, Nanjing Agricultural University, Nanjing, China; 2 Institute for Cell and Molecular Biosciences, The Medical School, University of Newcastle, Newcastle, United Kingdom; Institut National de la Santé et de la Recherche Médicale, France

## Abstract

There is controversy in the literature over whether the selenium (Se) influences cellular immune responses, and the mechanisms possibly underlying these effects are unclear. In this study, the effects of Se on T-cell proliferation and IL-2 production were studied in primary porcine splenocytes. Splenocytes were treated with different mitogens in the presence of 0.5–4 µmol/L sodium selenite. Se significantly promoted T-cell receptor (TCR) or concanavalin A (ConA)-induced T-cell proliferation and IL-2 production but failed to regulate T-cell response to phytohemagglutinin (PHA). In addition, Se significantly increased the levels of cytosolic glutathione peroxidase (GPx1) and thioredoxin reductase 1 (TR1) mRNA, the activity of GPx1 and the concentration of reduced glutathione (GSH) in the unstimulated, or activated splenocytes. These results indicated that Se improved the redox status in all splenocytes, including unstimulated, TCR, ConA and PHA -stimulated, but only TCR and ConA-induced T-cell activation was affected by the redox status. N-acetylcysteine (NAC), a pharmacological antioxidant, increased T-cell proliferation and IL-2 production by TCR and ConA stimulated splenocytes but had no effect on the response to PHA in primary porcine splenocytes confirming that PHA-induced T-cell activation is insensitive to the redox status. We conclude that Se promotes GPx1 and TR1 expression and increases antioxidative capacity in porcine splenocytes, which enhances TCR or ConA -induced T-cell activation but not PHA-induced T-cell activation. The different susceptibilities to Se between the TCR, ConA and PHA -induced T-cell activation may help to explain the controversy in the literature over whether or not Se boosts immune responses.

## Introduction

Selenium (Se) is an essential trace element for mammals and many other forms of life [1]. The biological effects of Se are mainly exerted through its incorporation into selenoproteins as the amino acid, selenocysteine (Sec). The well-characterized selenoproteins include enzymes such as cytosolic glutathione peroxidase (GPx1) and thioredoxin reductases (TR), which have Sec residues in the catalytic centers and function as antioxidant enzymes [1], [2]. Several of these and other less well-characterized selenoproteins have been shown to be expressed in nearly all tissues and cell types, including those involved in innate and adaptive immune responses [1], [3]–[5]. Selenoproteins are thought to play roles in the effects of altered Se status on immune responses [5], [6].

Severe Se deficiency is associated with numerous diseases such as Keshan disease and Kashin-Beck disease in humans [4], “white muscle disease” in calves and lambs [7]. In addition, Se deficiency has been implicated in accelerated disease progression and poorer survival among populations infected with human immunodeficiency virus [8]. However, effects of more subtle changes in Se status are less well defined and definition of the functions and underlying mechanisms is lacking. Se has been shown to cause immune response promotion [9] and cytokine production [10], and in contrast, immune response inhibition [11], and induction of cell death or apoptosis [12]–[14].

Although a number of studies have explored Se biology in T-cells and other immune cells [2], [9], [15], gene expression of selenoproteins and their responses to Se status remain largely unclear in the pig. Studies of the pig as an experimental model have commonly been consigned to specialist animal science journals. In fact, pigs offer a great physiological similarity to humans, with regards to food intake, energy expenditure for body size, body proportion and innate or adaptive immune response [16]–[19]. With the completion of the genome sequence and the characterization of many key regulators and markers, the pig will emerge as a tractable model of human nutrition and immunity. The present study was thus designed to evaluate the effects of Se, supplied as sodium selenite, on the different mitogen-induced T-cell proliferation, IL-2 production, levels of GPx1 and TR1 mRNA, as well as GPx1 activities and intracellular content of GSH in primary porcine splenocytes.

## Results

### Se supplementation promoted ConA-induced proliferation, but not PHA-induced proliferation

**Figure 1:**
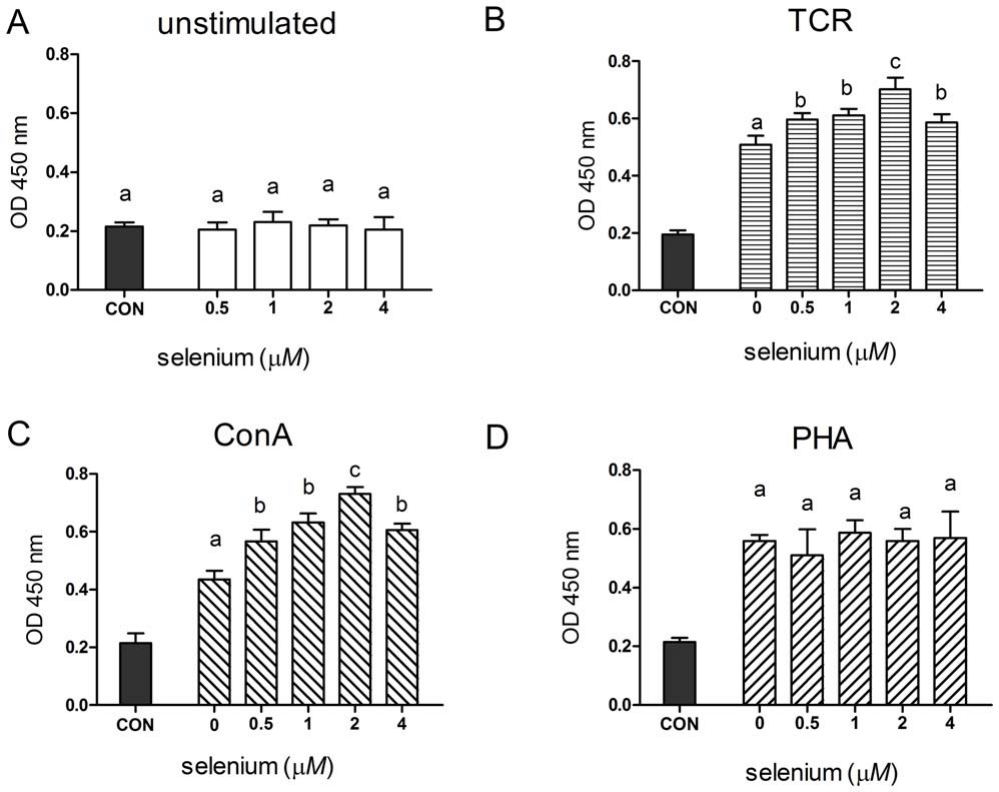
Selenium supplementation promoted TCR and ConA-induced T-cell proliferation, but not unactivated and PHA-induced proliferation. Primary porcine splenocytes (5×10^5^ cells) were treated with different concentrations of sodium selenite for 48 h in the absence (A) or presence of anti-CD3 (B), ConA (C) or PHA (D). T-cell proliferation was analyzed using a WST-8 Cell Counting Kit-8. Cells without any stimulus were used as control (CON). Data represent mean ± S.E. of two independent experiments, each measured in quadruplicate. Mean values without common letters within a given mitogen were significantly different (*P*<0.05).

To assess the effect of Se on the proliferative ability of lymphocytes, we first evaluated the effect of various concentrations of sodium selenite (0.5–4 µmol/L) on both unstimulated and activated splenocytes. Selenite significantly promoted T-cell receptor (TCR)-induced and concanavalin A (ConA)-induced proliferation at all concentrations (*P*<0.05), with a maximal increase at 2 µmol/L (Fig. 1B, C). In contrast, unstimulated or phytohemagglutinin (PHA)-induced T-cell proliferation was not affected by selenite within the range of concentrations tested (Fig. 1A, D).

**Figure 2:**
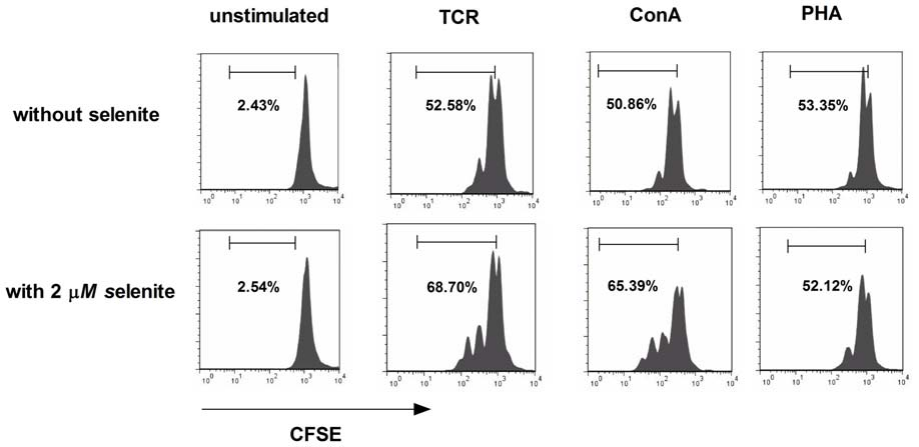
CD3^+^ T cell proliferation monitored using CFSE labeling. CFSE labeled splenocytes (2×10^6^ cells/well) were treated with or without 2 µmol/L of sodium selenite in the absence or presence of anti-CD3, ConA or PHA, and proliferation was analyzed at 48 h of culture. Cells were stained with anti-CD3-PEcy5. CD3^+^ T cells from the entire well were analyzed for proliferation by flow cytometry. The percentage of proliferating cells for each culture is indicated. A representative experiment from two separate experiments is shown.

In order to assess whether the proliferation observed by the cell counting assay was due to increased T cell proliferation or increased proliferation of other cell type, we assessed CD3^+^ T-cell proliferation by carboxyfluorescein diacetate, succinimidyl ester (CFSE) labeling of splenocytes and following anti-pig-CD3-PEcy5 staining. The T-cell proliferation measured by CFSE showed a similar pattern to that of water soluble tetrazolium salt-8 (WST-8, containing [2-(2-methoxy-4-nitrophenyl)-3- (4-nitrophenyl) 5-(2,4-disulfophenyl)-2H-tetrazolium, monosodium salt]). Total CD3^+^ T-cells proliferated more in the selenium treated group than in the control group in TCR or ConA stimulated splenocytes (53% versus 69%; 51% versus 65%, Fig. 2), but the percentage of proliferating cells did not differ in the unstimulated and PHA-stimulated groups (2.4% versus 2.5%; 53% versus 52%, Fig. 2).

### Effects of Se supplementation on IL-2 production in splenocytes

**Figure 3:**
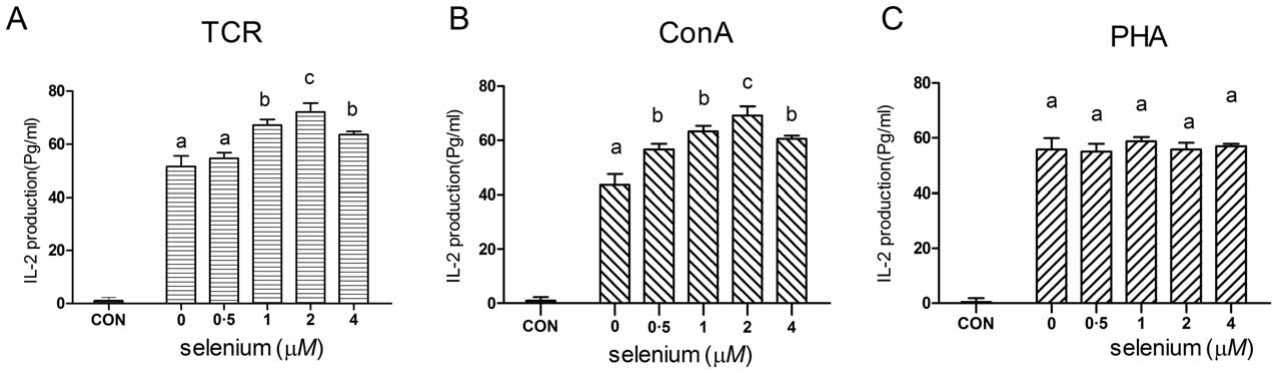
IL-2 production in splenocytes treated with different concentrations of sodium selenite. Primary porcine splenocytes were treated with different concentrations of sodium selenite for 48 h in the presence of anti-CD3 (A), ConA (B) or PHA (C). Cells without any stimulus were used as control (CON). Then the cell supernatants were collected and IL-2 concentration was determined by ELISA. Data represent mean ± S.E. of two independent experiments, each measured in triplicate. Mean values without common letters within a given mitogen were significantly different (*P*<0.05).

Production of IL-2 is a hallmark of activated T cells [20]. As shown in Fig. 3, selenite significantly promoted the stimulated production of IL-2 in splenocytes stimulated with TCR or ConA at all concentrations (*P*<0.05) (Fig. 3A, B). In contrast, the stimulated production of IL-2 in splenocytes stimulated with PHA was not affected by selenite within the range of concentrations tested (Fig. 3C). Furthermore, the stimulated production of IL-2 in unactivated splenocytes treated with varying concentrations of selenite almost the same as those produced in resting cells (data not shown).

### Effect of Se supplementation on selenoenzyme mRNA levels

**Figure 4:**
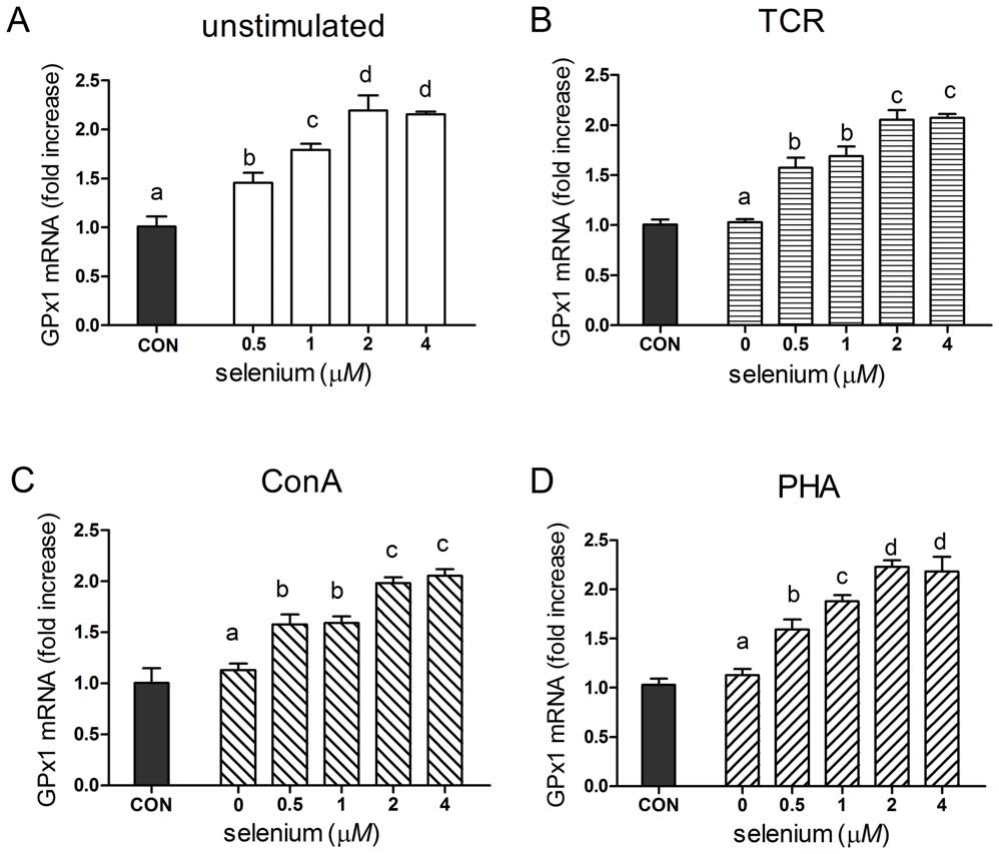
Effects of sodium selenite on GPx1 mRNA levels in porcine splenocytes. Primary porcine splenocytes (2×10^6^ cells) were treated with different concentrations of sodium selenite for 48 h in the absence (A) or presence of anti-CD3 (B), ConA (C) or PHA (D). Cells without any stimulus were used as control (CON). GPx1 mRNA was measured by quantitative real-time RT-PCR, and the ratio of the level of GPx1 mRNA to that of the β-actin internal control was used for statistical comparison. Data represent mean ± S.E. of two independent experiments, each measured in triplicate. Mean values without common letters within a given mitogen were significantly different (*P*<0.05).

GPx1 and TR1 mRNA levels in stimulated splenocytes were determined by quantitative RT-PCR. As shown in Fig. 4, Se supplementation significantly increased the GPx1 mRNA levels in all the groups (*P*<0.05) with both the pattern and the magnitude of the GPx1 mRNA increase in cells stimulated with TCR, ConA or PHA being similar to that of unstimulated cells (Fig. 4). At doses of 0.5–2 µmol/L, a significant dose-dependent increase in GPx1 mRNA level was measured in all groups treated with selenite (*P*<0.05) (Fig. 4). The level of GPx1 mRNA reached a plateau at 2 µmol/L of selenite supplementation (approximately 2-fold higher than in control group without added selenite) in all unstimulated, TCR, ConA or PHA-stimulated splenocytes.

**Figure 5:**
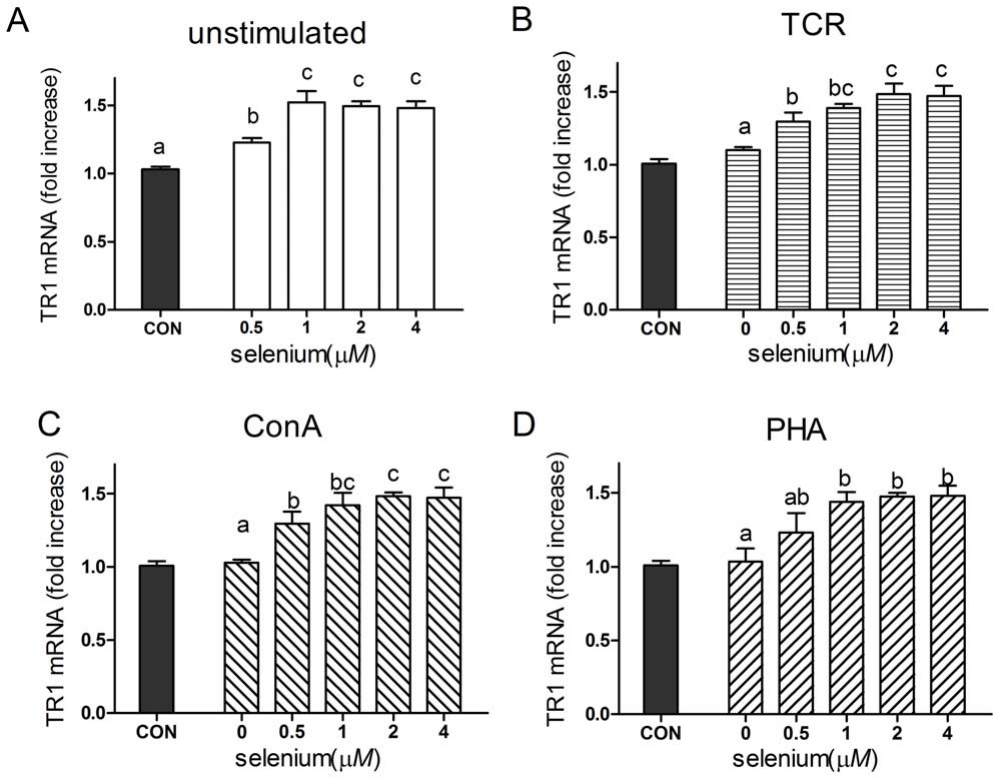
Effects of Se supplementation on TR1 mRNA level in porcine splenocytes. Primary porcine splenocytes (2×10^6^ cells) were treated with different concentrations of sodium selenite for 48 h in the absence (A) or presence of anti-CD3 (B), ConA (C) or PHA (D). Cells without any stimulus were used as control (CON). TR1 mRNA was measured by quantitative real-time RT-PCR, and the ratio of the level of TR1 mRNA to that of the β-actin internal control was used for statistical comparison. Data represent mean ± S.E. of two independent experiments, each measured in triplicate. Mean values without common letters within a given mitogen were significantly different (*P*<0.05).

The level of TR1 mRNA reached a plateau at 1 µmol/L of selenite supplementation (about 1.5-fold) in all unstimulated, TCR, ConA or PHA -stimulated splenocytes (Fig. 5). The TR1 mRNA expression patterns were similar to the GPx1 mRNA expression patterns, but the absolute magnitudes of the differences were lower. Levels of GPx4 mRNA did not differ in unstimulated and activated cells supplied with selenite (data not shown).

### Effect of Se supplementation on GPx1 activity

**Figure 6:**
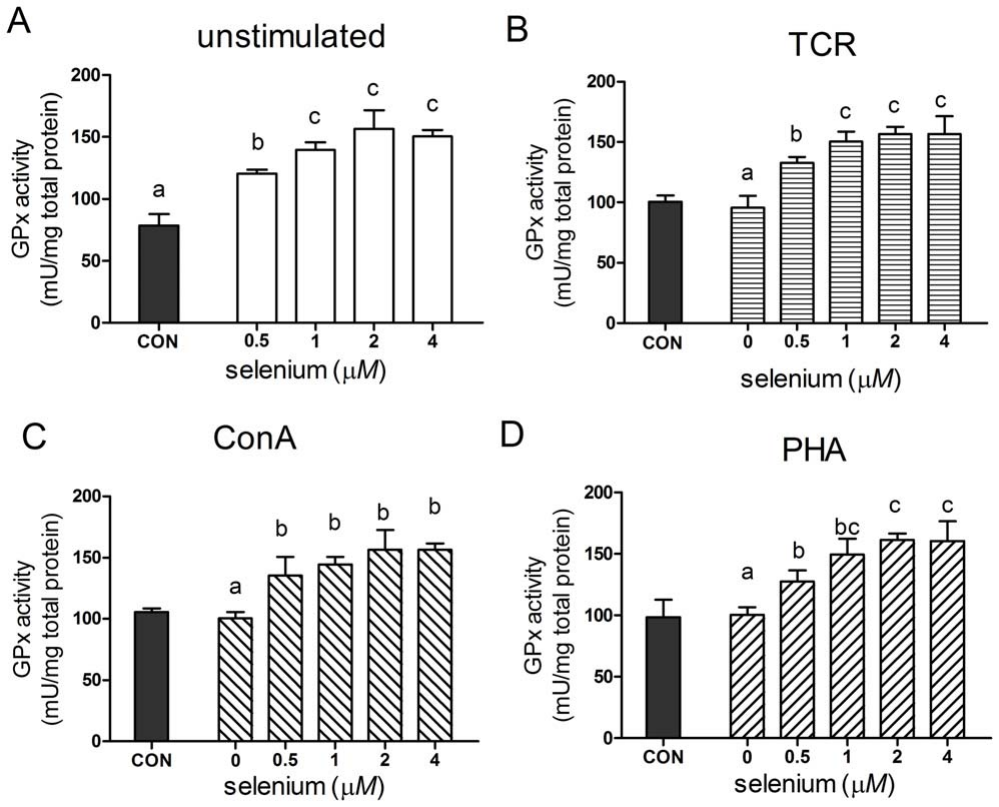
GPx1 activity in splenocytes treated with different concentrations of sodium selenite. Primary porcine splenocytes were treated with different concentrations of sodium selenite for 48 h in the absence (A) or presence of anti-CD3 (B), ConA (C) or PHA (D). **Cells without any stimulus were used as control (CON).** The activity of GPx1 in splenocyte cytosol was measured spectrophotometrically and expressed as units per gram of protein. Data represent mean ± S.E. of two independent experiments, each measured in triplicate. Mean values without common letters within a given mitogen were significantly different (*P*<0.05).

Activity of GPx1 in both unstimulated and activated splenocytes were significantly affected (*P*<0.05) by Se supplementation (Fig. 6). In the cells stimulated with PHA, ConA or TCR, both the pattern and the magnitude of the increases were similar to that of unstimulated cells (Fig. 6). At doses of 0.5–2 µmol/L, a significant dose-dependent increase in GPx1 activity was measured in all groups treated with selenite (*P*<0.05) with activity reaching a plateau of approximately 1.5-fold increase at 2 µmol/L of selenite supplementation in all unstimulated, TCR, ConA or PHA -stimulated splenocytes. The activity of GPx1 showed a similar pattern to that of GPx1 mRNA.

### Changes in intracellular GSH concentration

**Figure 7:**
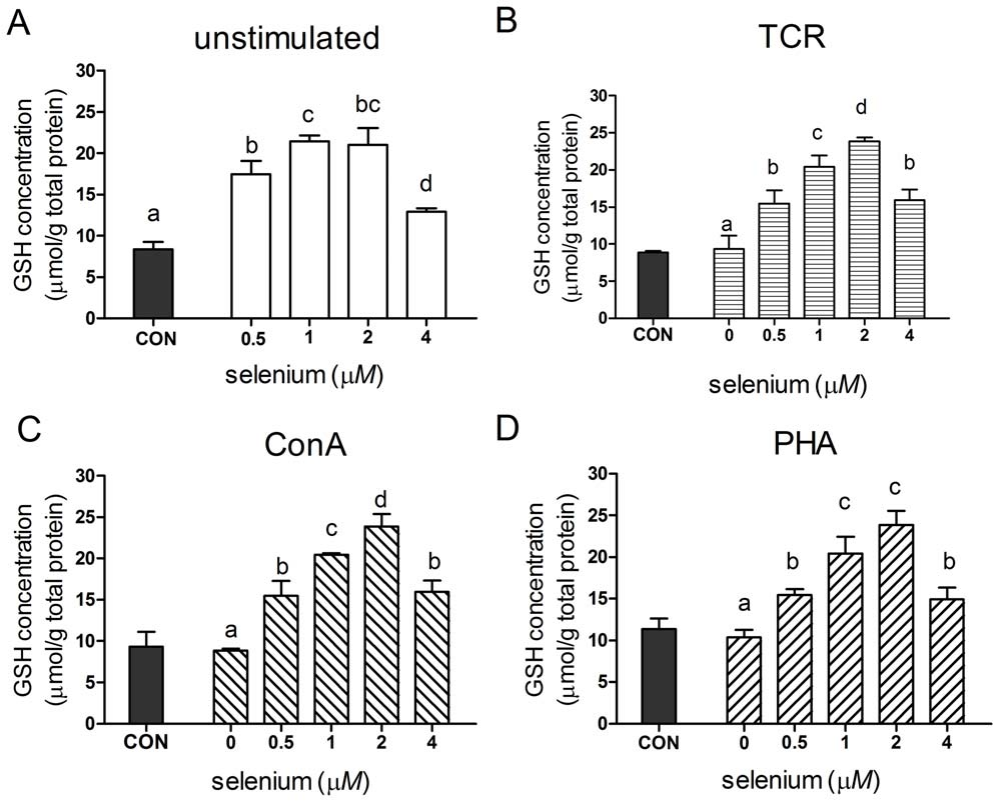
GSH levels in splenocytes treated with different concentrations of sodium selenite. Primary porcine splenocytes were treated with different concentrations of sodium selenite for 48 h in the absence (A) or presence of anti-CD3 (B), ConA (C) or PHA (D). Cells without any stimulus were used as control (CON). Total GSH concentration in splenocyte cytosol was measured spectrophotometrically. Data represent mean ± S.E. of two independent experiments, each measured in triplicate. Mean values without common letters within a given mitogen were significantly different (*P*<0.05).

To assess cellular redox tone, GSH levels were measured in the primary porcine splenocytes following Se supplementation. In all Se-treated splenocytes, intracellular concentrations of GSH were significantly enhanced (*P*<0.05). The maximal increases of GSH level were observed in unstimulated and activated splenocytes incubated with 2 µmol/L sodium selenite (Fig. 7). After reaching a maximal level, further Se supplementation led to a reduction of GSH concentration (Fig. 7).

### The susceptibility of different mitogen-induced T-cell proliferation to altered redox status

**Figure 8:**
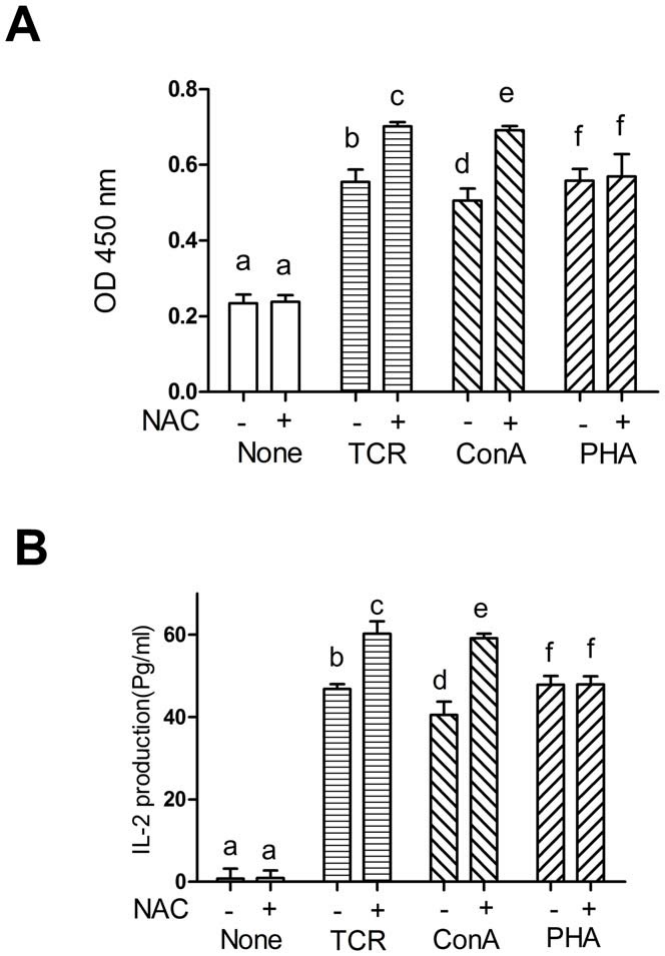
Effects of N-acetylcysteine (NAC) on mitogen-induced proliferation (A) and IL-2 production (B) of porcine splenocytes. Primary porcine splenocytes were either unstimulated (None) or stimulated as indicated with anti-CD3, ConA or PHA in the absence or presence of 5 mmol/L of NAC. Data represent mean ± S.E. of two independent experiments, each measured in quadruplicate. Mean values without common letters within a given mitogen were significantly different (*P*<0.05).

To determine whether change of redox status differentially affected T-cell proliferation and IL-2 production following stimulation by different mitogens, splenocytes were stimulated with or without the addition of the pharmacological antioxidant N-acetylcysteine (NAC), a thiol containing antioxidant and a precursor of GSH [21]. The addition of 5 mmol/L of NAC enhanced the TCR and ConA-induced T-cell proliferation and IL-2 production (*P*<0.05) (Fig. 8A, B), and the effects of Se supplementation on ConA-induced proliferation would be eliminated by pretreating the splenocytes with NAC (Fig. S1). In contrast, NAC did not affect proliferation and IL-2 production in unstimulated or PHA-stimulated splenocytes (Fig. 8A, B).

## Discussion

Previous work has suggested that Se supply affects T-cell function [9]. The data from the present study extend these observations by demonstrating that proliferation and IL-2 production of T-cells following activation by different mitogens varies in response to Se supplementation. Se supplementation promoted T-cell activation in primary porcine splenocytes when anti-CD3 mAb or ConA were used as a mitogen. The effect was specific, since no increase was observed in either basal or PHA-induced activation.

To our knowledge, there are no previous reports showing such a differential effect of Se on T lymphocytes from normal individuals. However, earlier findings that differences in responses of T-cells to an anti-CD3 mAb (OKT3) and PHA differed between phenylketonuria (PKU) patients of high and low Se status [22] are compatible with the present data. The T-cell responses to TCR stimulation were significantly reduced in PKU patients with reduced serum Se compared with a normal group and with a group of PKU patients whose serum Se was normal, but the T-cell response of the Se-deficient group to PHA did not differ from that of the normal controls or from that of Se-normal PKU patients. These data, together with the present observations suggest that both in humans and in pigs T-cell activation induced by different mitogens differ in sensitivity to Se status.

The biological effects of Se are mainly exerted through its incorporation into selenoproteins [23] and a recent study demonstrated that a complete lack of selenoproteins in T-cells led to decreased pools of mature T-cells and defective T-cell activation [15]. Therefore it is possible that the observed differences in the present study could have resulted from differences in the pattern of selenoprotein expression and/or change of redox status caused by alterations in Se status; TCR or ConA-induced T-cell activation could cause a greater increase than PHA-induced activation in selenoprotein expression in the presence of Se supplementation. However, this did not appear to be the case for either GPx1 or TR1 since the increase in GPx1 and TR1 mRNA levels, and GPx1 activity, after Se supplementation was similar in unstimulated, TCR, ConA or PHA-stimulated splenocytes.

GPx1 activity and mRNA level are known to decrease dramatically under conditions of Se deficiency, and to increases during Se repletion, thus making GPx1 a useful biomarker for Se status [24]. Previous studies indicated that Se supplementation led to a significant increase in GPx activity in T-cell and other tissues [9], [25], [26]. A recent study by Romanowska et al. [27] also indicated that the mRNA level and activity of GPx1 in HPL1D cells exhibited maximal levels at 10 nM selenite supplementation, without further change at higher concentrations. In the present study, GPx1 mRNA level and activity showed similar responses to Se supplementation among mitogens and different Se concentrations. Although selenite significantly increased mRNA level and activity of GPx1 in both unstimulated and mitogen-stimulated splenocytes, the effects of Se were quantitatively smaller for activity compared with mRNA level. This result is consistent with Se affecting GPx protein through at least two mechanisms, mRNA stability, and level of Sec regulating translation [27]. We have previously observed a similar differential effect in bovine hepatocytes [28], when GPx1 mRNA was increased 3- to 6-fold, while GPx1 activity was 1.3- to1.5-fold higher.

The expression of TR1 was also directly dependent on Se concentration. TR1 mRNA reached a plateau in all splenocytes supplied with selenite at a dose of 1 µmol/L, but TR1 levels are only modestly increased when compared with GPx1 mRNA. In contrast, GPx4 mRNA remained unchanged by selenite supplementation (data not shown), as was also observed in rat liver [16]. Overall, these data are in agreement with previous studies that found GPx1 mRNA to be more dramatically regulated by Se status than many other selenoprotein mRNAs [29]. This hierarchy of Se regulation of the various selenoproteins mRNAs is not fully understood, although nonsense-mediated decay (NMD) may play a role [30].

Given the integral role of oxidative stress in modulating the activation of T-cells into effectors [31], GSH levels were measured as an indicator of redox status in the primary porcine splenocytes after Se supplementation. GSH, a ubiquitous thiol-containing tripeptide, is the major intracellular antioxidant with multiple biological functions, including the maintenance of thiol group of the cysteine residue [32] and the reduced form of many other molecules [33]. Intracellular GSH concentration in unstimulated, or mitogen-stimulated splenocytes was found to increase after selenium supplementation. Since glutathione is closely involved in Se metabolism and bioactivity, acting as a co-substrate of GPx and playing an important role in the scavenging of reactive oxygen species, free radicals and reactive metabolites that may be generated under various conditions [28], this may be attributable to the fact that concentrations of GSH rise during lower oxidative stress following Se supplementation [9]. Additionally, selenite undergoes thiol-dependent reduction to form selenide before being incorporated into selenoproteins [13], and this may well explain why selenite at the higher concentration led to a reduction of GSH concentration. Also, the patterns and the magnitude of GSH concentration in splenocytes from unstimulated, and all three mitogen-stimulated groups were similar after treatment with selenite.

Since the results of intracellular GSH concentrations indicated that Se improved the redox status in all splenocytes treated with sodium selenite, but only TCR and ConA-induced T-cell proliferation and IL-2 production was affected by the redox status, it seems likely that the difference between the TCR-induced, ConA-induced and PHA-induced T-cell activation to Se supplementation is not due to a variation in selenoproteins expression or antioxidative capacity but rather due to a different susceptibility to redox status. The finding that NAC, a pharmacological antioxidant, promoted T-cell response to TCR stimulation and ConA, but not the PHA response in primary porcine splenocytes further argues for this hypothesis. Previous studies demonstrated that TCR signal strength in T cells is affected by Se supplementation and selenoproteins [9], [15]. ConA, a T-cell specific mitogen and a known TCR/CD3 complex ligand, activate T-cells predominantly via a specific component of the TCR/CD3 complex [34], [35]. However, the mitogenic lectin PHA has been reported to modulate T-cell proliferation via the antigen (TCR) and alternate (CD2) pathways [36]. Although previous studies have shown that Ca^2+^ mobilization, Jun N-terminal kinase (JNK), and p38 MAP kinase signaling differ in ConA and PHA-induced T-cell activation [37], [38], exact molecular targets in T-cells that contribute to this different susceptibility to redox status remain to be identified. The mechanisms by which oxidative stress and selenoproteins modulate these different pathways are relatively unexplored but undoubtedly important areas for future investigation.

Taken together, our results show that Se promoted T-cell response to TCR stimulation and ConA, but failed to regulate PHA-induced T-cell proliferation and IL-2 production. Se status has been shown to regulate GPx1 and TR1 mRNA in all modes of T-cell activation, and also has direct effects on GPx1 activity and cellular redox status, which contribute to promote T-cell response to TCR stimulation or ConA. The data suggest that the difference among the TCR, ConA and PHA-induced T-cell activation to Se supplementation is mostly due to a different susceptibility to redox status. The notion that the roles of Se in the activation of T-cells differ in T-cells stimulated with different mitogens may help to explain studies showing that Se exerts differential influences on various types of immune response [6], [39], and ultimately the capability of Se to boost immunity.

## Materials and Methods

### Animals and cell culture

Normal Meishan pigs aged 30 days and weighing 8±2 kg were purchased from the breeding center of Jiangsu Polytechnic College of Agriculture and Forestry. All pigs were fed diets containing 0.14 mg/kg Se. The experimental use of animals and procedures followed were approved by the Nanjing Agricultural University Animal Care Committee.

Pigs were killed and then the spleens were removed under sterile conditions, disrupted mechanically, and the splenocytes were resuspended in Hanks' solution and carefully layered on the surface of lymphocyte separation medium (Tianjin Hematology Institute, Chinese Academy of Medical Sciences, Tianjin, China). After centrifugation at 1200×g for 20 min, the white cloudy band of splenocytes was collected. The splenocytes were washed twice with RPMI-1640 media without fetal bovine serum (FBS) and then maintained in RPMI-1640 medium supplemented with 10% FBS in the presence of penicillin/streptomycin at 37°C, 5% CO_2_ and at a density of 5×10^6^cells/ml. Viability of isolated splenocytes was assessed by 0.4% trypan blue dye exclusion.

### Stimulation and proliferation assays

T-cell mitogenic responses were analyzed using a WST-8 Cell Counting Kit-8 (Beyotime, Jiangsu, China) as described previously [40]. In pilot experiments we tested each mitogen with varying concentrations to obtain a sub-optimal concentration of each mitogen and chose a similar OD value to ensure the stimulatory effect of each mitogen were comparable (data not shown). Briefly, 5.0×10^5^ cells suspended in RPMI-1640 medium (200 µl) containing 10% FBS were seeded in 96-well plates and treated with 0, 0.5, 1, 2 or 4 µmol/L of sodium selenite (Sigma) in absence (controls) or presence of 5 µg/ml ConA (Sigma), 10 µg/ml PHA (Sigma) or 5 ug/ml stimulatory anti-pig-CD3 mAb (clone PPT3, Abcam). After incubation for 48 h, CCK-8 solution (20 µl) was added to each well, the cells were incubated at 37°C for 3 h and then the absorbance at 450 nm was measured. In experiments when cells were treated with NAC, 5 mmol/L NAC was added in RPMI-1640 medium in absence or presence of ConA, PHA or anti-pig-CD3 mAb.

### CFSE-labeling of splenocytes

Splenocytes were rested overnight in RPMI1640 medium at 2×10^6^ cells/ml with analysis of unstimulated samples on day 1. For assessment of T cell proliferation, an equal volume of 10 mM CFSE (Invitrogen) was added to the unstimulated splenocytes suspension. Samples were vortexed and incubated at 37°C in the dark for 10 min. To quench the CFSE reaction, a double volume of ice cold FBS was added. Samples were again vortexed and incubated at room temperature in the dark for 5 minute. Splenocytes were washed twice with warm RPMI 1640 medium with 25 mM HEPES supplemented with 10% FBS to remove excess CFSE. 2×10^6^ CFSE stained cells were seeded in a 24-well culture plate (Costar) in RPMI-1640 medium to a final volume of 1 ml. Stimulated samples were treated with or without 2 µmol/L of sodium selenite in absence (controls) or presence of 5 µg/ml ConA, 10 µg/ml PHA or 5 ug/ml stimulatory anti-CD3 mAb. Unstimulated samples were used as negative controls. Plates were incubated at 37°C, 5% CO_2_ for 48 h. Each culture was performed in duplicate.

### Flow cytometry

Cultures were analyzed for proliferation after 48 h. Cells were stained with anti-CD3-PEcy5 (clone PPT3, Abcam). CD3^+^ T-cells from the entire well were analyzed for proliferation by flow cytometry. Flow cytometry was performed using FACSCalibur (BD Biosciences) flow cytometers with acquisition enabled by CellQuest Pro software (BD Biosciences). Color compensation was achieved using an appropriate single fluorochrome-labelled sample. Data was analyzed using FlowJo 7.6.5 (TreeStar).

### Determination of IL-2 in splenocytes

Splenocytes (2×10^5^ cells/well) were seeded in 96-well plates and treated with varying concentrations of sodium selenite in absence (controls) or presence of anti-CD3 mAb, ConA or PHA for 48 h. The cell supernatants were then collected and assayed for IL-2 by ELISA (R&D Systems). In experiments when cells were treated with NAC, 5 mmol/L NAC was added in RPMI-1640 medium in absence or presence of anti-CD3 mAb, ConA or PHA.

### Real-time RT-PCR

2.0×10^6^ cells were seeded in 6-well plates and cultured as described above. Total RNA was isolated from the splenocytes using the RNAiso Plus (TaKaRa, Dalian, China) according to the manufacturer's protocol. The RNA pellet was dissolved in diethyl pyrocarbonate-treated water, quantified by measurement of the absorbance at 260/280 nm and stored at −70°C prior to cDNA synthesis. First-strand cDNA was synthesized from 1 µg of total RNA using Oligo dT primers and RTase M-MLV (TaKaRa, Dalian, China) according to the manufacturer' instructions. Synthesized cDNA was diluted 10 times with sterile water and stored at −20°C before use. Primer Premier software (PREMIER Biosoft International, Palo Alto, CA, USA) was used to design specific primers for β-actin, GPx1 and TR1 based on known porcine sequences (Table 1). Quantitative real-time PCR was carried out as previously described [28], with some modifications. In brief, reactions were performed in a 25-µl reaction mixture containing 12.5 µl of 2×SYBR Green I PCR Master Mix (TaKaRa, Dalian, China), 10 µl of either diluted cDNA or plasmid standard, 1 µl of each primer (10 µmol/L) and 0.5 µl of PCR-grade water. Reactions were followed in an ABI PRISM 7300 Detection System (Applied Biosystems, USA) which consisted of a 95°C step for 30 s followed by 40 cycles consisting of 95°C for 5 s, 60°C for 31 s. A dissociation curve was run for each plate to confirm the production of a single product. The calculation of the number of copies of each sample was performed from the respective standard curve using the 7300 system software. The ratio of the level of GPx1 and TR1 mRNA to that of the β-actin internal control was used for statistical comparison of the different treatments.

**Table 1:** Primers used for quantitative real-time PCR.

Primers	Nucleotide sequence(5′ –3′)	Target gene	GenBank accession no.	PCR fragment length (bp)
β-Actin-F (forward)	CTGCGGCATCCACGAAACT	β-Actin	DQ845171.1	147
β-Actin-R (reverse)	AGGGCCGTGATCTCCTTCTG			
GPx1-F (forward)	TGGGGAGATCCTGAATTG	GPx1	NM_214201	172
GPx1-R (reverse)	GATAAACTTGGGGTCGGT			
TR1-F (forward)	CCCTGGTGACAAAGAGTA	TR1	NM_214154	184
TR1-R (reverse)	GTCCTGGTCAAATCCTCT			

### GPx1 activity

Cells were harvested into 1 ml of cold Tris buffer (20 mM Tris–HCl, pH 7.5, 2 mM EDTA and 0.1% peroxide-free Triton X-100). Splenocyte lysates were then prepared by sonication for 30 s in ice-cold water and then centrifuged at 12,000×g for 15 min at 4°C. The supernatants were aliquoted, stored at −20°C for subsequent analysis and GPx1 activity was measured according to the method of Lei et al. [41], with tert-butyl hydroperoxide (t-Bu-OOH) as the peroxide substrate. Briefly, 50 µl of splenocyte lysate was transferred to a 3-ml quartz cuvette containing 1900 µl of the reaction mixture [50 mM Tris–HCl, pH 7.5, 2 mM EDTA, 0.1 mM reduced nicotinamide adenine dinucleotide phosphate (NADPH), 2 mM GSH, 1 mM NaN_3_ and 0.9 U of GSH reductase (Sigma)]. The reaction mixture was preincubated for 3 min at 25°C, and the reaction was initiated by adding 50 µl of t-Bu-OOH (8 mM). The rate of oxidation of NADPH was monitored in a spectrophotometer at 340 nm for 5 min at 25°C. The nonenzymatic reaction rate was determined by substituting water for the splenocyte lysate and recording the decrease in NADPH absorbance. One unit of enzyme activity was defined as 1 µmol of NADPH oxidized per minute under these conditions. The activity of GPx1 in splenocyte cytosol was expressed as units per gram of protein. Total protein concentration was determined with Bicinchoninic Acid assay (Beyotime Institute of Biotechnology, China). All samples were measured in duplicate.

### Reduced glutathione concentration

The concentration of GSH in splenocyte cytosol was measured as previously described [42] based on the reaction of 5,5′-dithiobis(2-nitrobenzoic acid) (Ellman's reagent) and GSH to 5-thio-2-nitrobenzoic acid, which absorbs light at a wavelength of 412 nm. The GSH concentrations were calculated from a standard curve prepared with pure GSH standards and were expressed as micromoles of GSH per gram of protein. All samples were assayed in duplicate.

### Statistical analysis

All statistical tests for comparison of means were performed using the SPSS 17.0 for Windows statistical software package (SPSS Inc., USA). A one-way ANOVA was used to determine effect of Se on outcomes for mitogen-stimulated cells with Tukey post test used to compare means of each groups within mitogens. For the experiment involving NAC and proliferation, intergroup differences were assessed by the Student's t test within mitogens. Differences were considered significant if *P*<0.05.

## Supporting Information

Fig. S1 **Effects of N-acetylcysteine (NAC) on ConA-induced proliferation for porcine splenocytes supplied with Se.** Primary porcine splenocytes were stimulated with ConA in the absence or presence of 5 mmol/L of NAC at first 24 h, washed with PBS once, and then treated with sodium selenite (2 µM) for another 24 h. Data represent mean ± S.E. of two independent experiments, each measured in quadruplicate. Mean values without common letters were significantly different (*P*<0.05).
(TIF)
