# Supplementary figures and images for: Selenium Promotes T-Cell Response to TCR-Stimulation and ConA, but Not PHA in Primary Porcine Splenocytes
Source: PLoS One. 2012 Apr 17;7(4):e35375. doi: 10.1371/journal.pone.0035375 (PMC3328446; doi:10.1371/journal.pone.0035375)

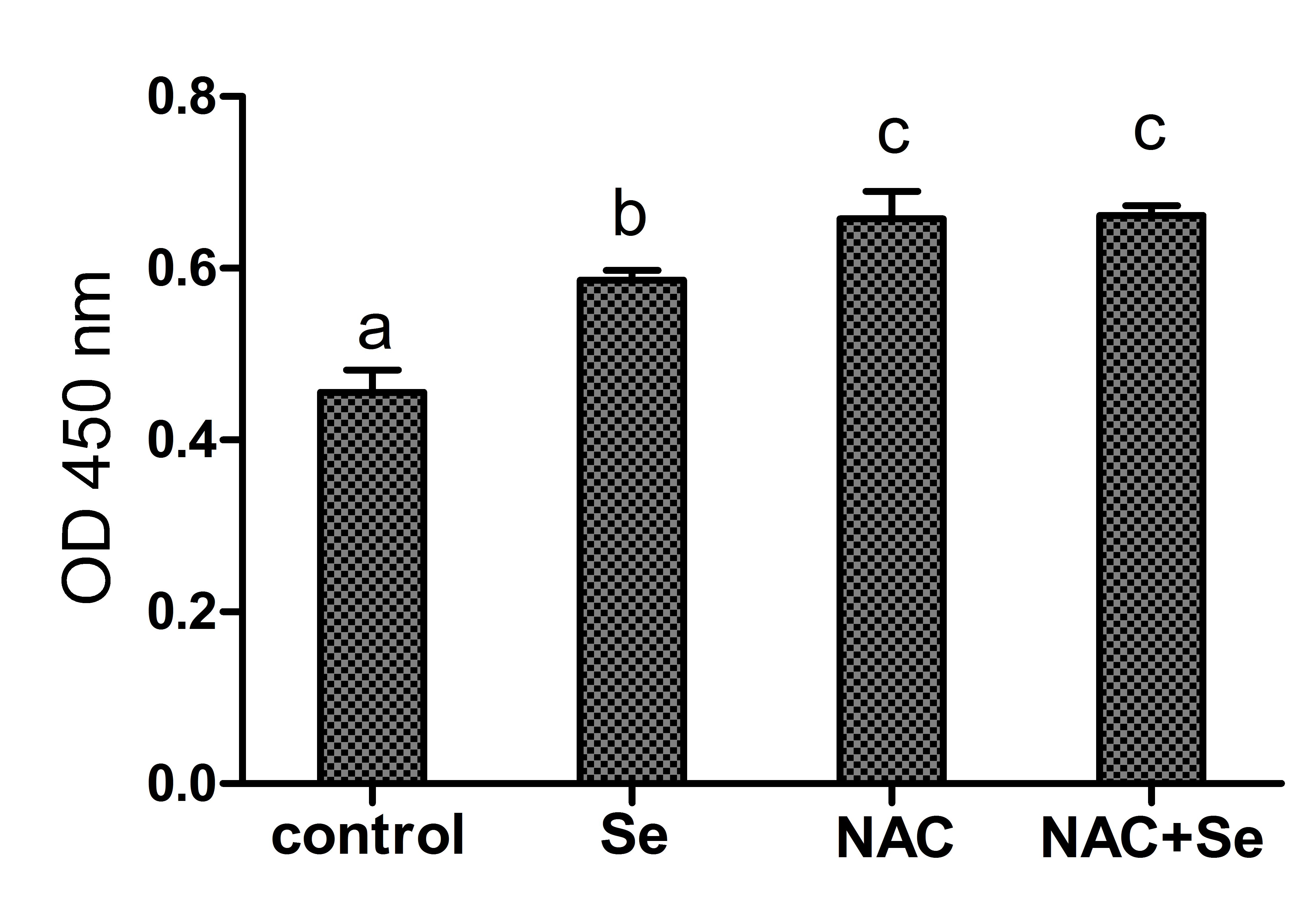

Supplement: Fig. S1 — Effects of N-acetylcysteine (NAC) on ConA-induced proliferation for porcine splenocytes supplied with Se. Primary porcine splenocytes were stimulated with ConA in the absence or presence of 5 mmol/L of NAC at first 24 h, washed with PBS once, and then treated with sodium selenite (2 µM) for another 24 h. Data represent mean ± S.E. of two independent experiments, each measured in quadruplicate. Mean values without common letters were significantly different (P<0.05). (TIF) [file pone.0035375.s001.tif]
